# Clinically constrained Bayesian network analysis of immune–metabolic factors associated with bone mineral density in a real-world orthopedic cohort

**DOI:** 10.3389/fsurg.2026.1934871

**Published:** 2026-08-19

**Authors:** Chong Zhao, Yuemei Hu, Zhenqi Zhu, Weiwei Xia, Haiying Liu

**Affiliations:** 1 Department of Spinal Surgery, Peking University People’s Hospital, Peking University, Beijing, China; 2 School of Computer Science, Georgia Institute of Technology, Atlanta, GA, United States

**Keywords:** Bayesian network, bone turnover markers, bridge mediation, clinical prior constraints, neutrophil-to-lymphocyte ratio, osteoporosis

## Abstract

**Objectives:**

Osteoporosis substantially influences surgical decision-making, implant fixation, fracture risk, and postoperative outcomes in orthopaedic practice. However, the systemic biological interactions underlying bone loss remain difficult to characterize using conventional statistical models. We aimed to integrate clinically constrained Bayesian network learning with routine biochemical data to identify immune–metabolic factors associated with bone mineral density (BMD) and to provide a clinically interpretable framework for bone health assessment.

**Methods:**

In a retrospective real-world orthopedic cohort of 2,880 individuals with concurrent DXA and biochemical data, we constructed a 20-node network comprising BMD, bone metabolism biomarkers, and immune-inflammatory, metabolic, renal, and endocrine indicators. Gaussian graphical models and prior-constrained Bayesian networks were applied, blacklisting edges that pointed to age or menopause status to enforce physiological plausibility. Bridge mediation analysis systematically scanned source—bone biochemistry—BMD pathways, using bootstrap confidence intervals. Analyses were repeated in the postmenopausal subgroup (*n* = 1,546).

**Results:**

Network hubs included parathyroid hormone (PTH), phosphorus, neutrophil-to-lymphocyte ratio (NLR), high-density lipoprotein (HDL), and triglycerides (TG). Prior constraints eliminated all prespecified prohibited edges. NLR–ALP–BMD emerged as the most robust negative bridging pattern (indirect effect for lumbar spine: −0.045; mediation proportion: 70.5% in the full cohort), with similar findings in postmenopausal women. NLR–PTH–BMD and NLR–phosphorus–BMD were also significant. The NLR–ALP–BMD indirect associations remained significant after hepatic-marker adjustment and in participants without elevated ALP or ALT, whereas the ALB–ALP–BMD signal was attenuated and inconsistent across subgroups. Positive indirect effects involving glucose, TG, and uric acid may reflect unmeasured adiposity and DXA measurement artifacts.

**Conclusion:**

Clinically constrained network analysis identified immune–metabolic factors associated with bone mineral density and provided a biologically plausible framework for interpreting bone–immune–metabolic interrelationships in orthopedic patients.

## 1 Introduction

Osteoporosis represents a major challenge in orthopedic and spine surgery because reduced bone quality adversely affects fracture risk, implant fixation, spinal instrumentation stability, and postoperative recovery. Consequently, accurate assessment of bone health has become increasingly important in surgical practice. Although osteoporosis is traditionally regarded as a metabolic bone disease, accumulating evidence indicates that bone loss reflects systemic immune, endocrine, nutritional, and metabolic dysregulation rather than isolated skeletal remodeling. Age-related sex hormone withdrawal (menopause), remodeling of thyroid axis function, chronic low-grade elevation of inflammatory cytokines, and disturbances in calcium and phosphorus metabolism secondary to declining renal function do not act on bone in isolation; rather, they form complex compensatory, cascading, and feedback networks among systemic nodes (1, 2). The concept of immunoporosis has further established the central role of innate and adaptive immune cells in directly regulating osteoclast differentiation through the receptor activator of nuclear factor-*κ*B ligand (RANKL)/receptor activator of nuclear factor-*κ*B (RANK)/osteoprotegerin (OPG) axis, pro-inflammatory cytokine release, and macrophage metabolic reprogramming (3). This suggests that the development of osteoporosis, particularly in postmenopausal women, may not be a linear process driven by a single factor, but rather a network collapse event involving simultaneous destabilization of multiple systems.

Understanding this network structure is essential for identifying the core driving nodes responsible for bone loss. However, the multivariable regression paradigm widely adopted in traditional epidemiological and clinical studies has two fundamental limitations when addressing such systemic problems. The first is the collinearity problem: extensive biological coupling exists among metabolic, immune, and nutritional indicators. Including highly correlated variables in a regression model not only leads to unstable estimates, but more importantly, this collinearity itself carries information about system coupling; treating it simply as statistical noise to be eliminated discards the most valuable part of the network structure (4, 5). Recent literature on metabolomic network analysis has repeatedly emphasized that intrinsic correlations among metabolites are not statistical flaws, but rather biological signals reflecting underlying low-dimensional manifold structures and modular organization (6, 7). The second limitation is the unidentifiability of causal inference in cross-sectional data. In observational data at a single time point, the statistical association between a biomarker and BMD may represent a causal effect, reverse causation (where changes in bone mass alter the expression of the biomarker), or, more commonly, both are merely parallel products of age and menopausal status. Unconstrained data-driven algorithms, including widely used Bayesian network structure learning, can readily produce biologically implausible edges with reversed causality, such as BMD pointing to age or a biochemical marker pointing to menopausal status. The literature on causal inference methodology explicitly states that identifying confounders in cross-sectional data must rely on prior judgments about temporal sequence and physiological irreversibility among variables, rather than purely data-driven conditional independence tests (8).

In recent years, Gaussian graphical models (GGMs) and Bayesian networks have been introduced into osteoporosis and BMD research to characterize association networks between biomarkers and BMD (9, 10). GGMs estimate conditional dependencies among variables through partial correlation coefficients, thereby avoiding spurious associations driven by confounders in naive correlation networks, and have become a mainstream tool for network reconstruction in multi-omics and biomarker studies (11). In BMD research, Bayesian networks have been applied to infer directed causal relationships among genes from single-cell transcriptomic data and have successfully identified candidate driver genes within GWAS signals for human BMD (12). However, a critical element is generally missing from the existing literature: the incorporation of clinical expert prior knowledge to constrain network structure learning. When facing high-dimensional collinear data with limited sample sizes, the reliability of the edge set produced by purely data-driven structure learning is heavily dependent on the regularization strategy and scoring function of the algorithm, rather than on biological plausibility. One practical strategy is to explicitly incorporate prespecified clinical knowledge regarding physiologically implausible edge directions into structure learning.

Based on the above considerations, this study aims to provide a network analysis paradigm that improves the biological interpretability of the estimated network in cross-sectional observational data by applying clinical prior constraints. In a retrospective cross-sectional cohort of 2,880 individuals, we constrained network structure learning with clinical logic such as temporal sequence and physiological irreversibility, excluded associations inconsistent with prespecified physiological principles, and, after reconstructing the network topology, conducted dual mediation analyses in the entire cohort and the postmenopausal subgroup to explore potential biochemical bridges through which systemic immune-inflammation and nutritional status are indirectly associated with BMD through bone metabolism biomarkers.

## 2 Methods

### 2.1 Study design and data source

This study was a single-center retrospective cross-sectional analysis. Data were obtained from the Hospital Information System (HIS) of Peking University People's Hospital. The study protocol was approved by the Ethics Committee of Peking University People's Hospital. Because data extraction and analysis involved only archived electronic medical records and did not require re-identification of patient identity information, the ethics committee granted a waiver of informed consent. Patient records containing both dual-energy x-ray absorptiometry (DXA) BMD measurements and available biochemical measurements linked at the patient level were extracted from the information system. The median absolute interval between DXA and the selected laboratory record was 1 day (IQR, 1–3 days), and 96.9% of records were obtained within 30 days of DXA. After cross-table linkage and deduplication, 2,880 individuals with complete lumbar spine and femoral neck T-score records were included as the analysis cohort.

### 2.2 Variable definition and data processing

The final network analysis included 20 nodes, consisting of 19 continuous variables and one binary clinical status variable. The continuous variables were lumbar spine T-score, femoral neck T-score, age, PTH, ALP, total calcium, phosphorus, NLR, white blood cell count, ALB, total cholesterol, HDL-C, triglycerides, glucose (Glu), uric acid (UA), thyroid-stimulating hormone (TSH), free thyroxine (FT4), ALT (alanine aminotransferase), and creatinine.

Menopause was coded as an electronic health record–derived indicator (1 = postmenopausal status explicitly recorded; 0 = no postmenopausal status recorded). Body mass index (BMI) had a missing proportion exceeding 50%, and the missingness pattern was clearly influenced by clinical collection procedures; C-reactive protein (CRP) had valid measurements in less than 5% of cases. Neither variable was included in the final main model, and no imputation was performed; instead, both were retained as unmeasured confounders at the interpretation level. The systemic immune-inflammatory status was represented by NLR as the core surrogate.

Continuous variables were first Winsorized at prespecified percentiles. Missing values were then imputed using multivariable iterative imputation implemented with the scikit-learn IterativeImputer algorithm, with the imputation models fitted in the full cohort. Continuous variables were subsequently standardized to Z-scores, whereas Menopause retained its binary 0/1 coding.

### 2.3 Network structure analysis

First, an undirected network was estimated from the 20-node matrix using a Gaussian graphical model (GGM). The GGM identifies partial correlation relationships between variables through non-zero elements in the precision matrix, thereby reducing naive correlations driven by common confounders. To obtain a stable sparse topology, we applied the Graphical Lasso with the regularization parameter set to alpha = 0.05. Subsequently, Louvain community detection was performed on the absolute partial correlation-weighted network to examine whether data-driven communities corresponded to *a priori* physiological modules. Because the network contains a binary state variable (Menopause), the GGM results were used primarily for conditional dependence screening and topological description rather than for strict multivariate Gaussian interpretation.

Second, Bayesian directed network structure learning was performed on the same variable set to explore directed dependencies. Structure learning used a hill-climbing search algorithm based on the Bayesian information criterion (BIC) score, with a maximum in-degree of 5. To reduce physiologically implausible edge directions in the cross-sectional data, we embedded clinical prior constraints: (1) no variable was allowed to point to age; (2) except for age, no variable was allowed to point to menopause status; (3) directed edges of any direction were prohibited between lumbar spine T-score and femoral neck T-score; (4) the edge from age to menopause status was enforced as mandatory. Structural stability was assessed by 30 bootstrap resamples with replacement, and edges with a support rate ≥ 50% were considered to have moderate or greater stability.

### 2.4 Bridge mediation and sensitivity analyses

The mediation analysis adopted a bridge mediation strategy. Candidate paths did not require each step of X-M-Y to be a stable edge in the directed acyclic graph (DAG); instead, based on the network analysis results and biological assumptions, we systematically traversed bridging patterns of source—bone biochemistry—BMD. The source nodes included NLR, ALB, TG, UA, HDL, total cholesterol (TC), Glu, and white blood cell count (WBC); the bridge nodes were restricted to PTH, Ca, P, and ALP; and the outcome nodes were lumbar spine T-score or femoral neck T-score. The product of coefficients approach was used, estimating the path coefficient from the exposure to the mediator (path a) and the path coefficient from the mediator to the outcome adjusting for the exposure (path b) via ordinary least squares regression; the indirect effect was a × b. Statistical inference for the indirect effect was based on 95% confidence intervals obtained from 500 bootstrap nonparametric resamples. A candidate bridging signal was considered directionally consistent when the confidence interval excluded zero and the indirect and direct effects had the same sign.

Covariate control applied a dynamic exclusion logic. The covariate pool for the entire cohort was set as age and menopause status; for the postmenopausal subgroup, the covariate pool was age only. When performing regression analysis for any given path, if the exposure or mediator variable itself was in the covariate pool, it was removed from the current covariate set to avoid having the same variable on both the exposure and covariate sides, which would render the parameter unidentifiable.

Sensitivity analyses of the NLR–ALP–BMD and ALB–ALP–BMD bridging patterns were performed using three nested models: the baseline covariate model, the baseline model with additional adjustment for ALT, and the baseline model with additional adjustment for ALT, GGT, and total bilirubin. Hepatic markers were included in both the mediator and outcome regression models. To ensure comparability before and after hepatic-marker adjustment, all nested models were fitted in the same participants with complete hepatic-marker data (full cohort, *n* = 2,178; postmenopausal subgroup, *n* = 1,251).

Subgroup analyses were defined using original, non-imputed laboratory values. The prespecified primary subgroups included participants with non-elevated ALP (≤125 U/L), non-elevated ALT (<40 U/L), or both conditions. Additional sensitivity definitions included normal-range ALP (45–125 U/L) and ALT <50 U/L. Because patient-specific laboratory reference intervals were unavailable in the extracted data, these prespecified adult thresholds were applied consistently.

After completing the whole-cohort analysis, we separately analyzed participants with an explicitly recorded postmenopausal status (*n* = 1,546) and repeated the directed network structure learning and bridge mediation analysis. Menopause was excluded from the subgroup models because it was constant, and age was retained as the covariate. Persistence of a bridging pattern in this subgroup was interpreted as an association observable among participants recorded as postmenopausal rather than as evidence of independence from estrogen-withdrawal mechanisms.

### 2.5 Statistical software

All data analyses and figure generation were performed using Python. Data preprocessing, GGM estimation, Bayesian network structure learning, and mediation analysis were implemented using the pandas, scikit-learn, pgmpy, and statsmodels packages, respectively; network visualization used matplotlib and seaborn. Statistical inference employed two-sided tests, and an indirect association was considered statistically significant if the bootstrap 95% confidence interval excluded zero.

## 3 Results

### 3.1 Cohort characteristics and global system interconnections

**Figure 1:**
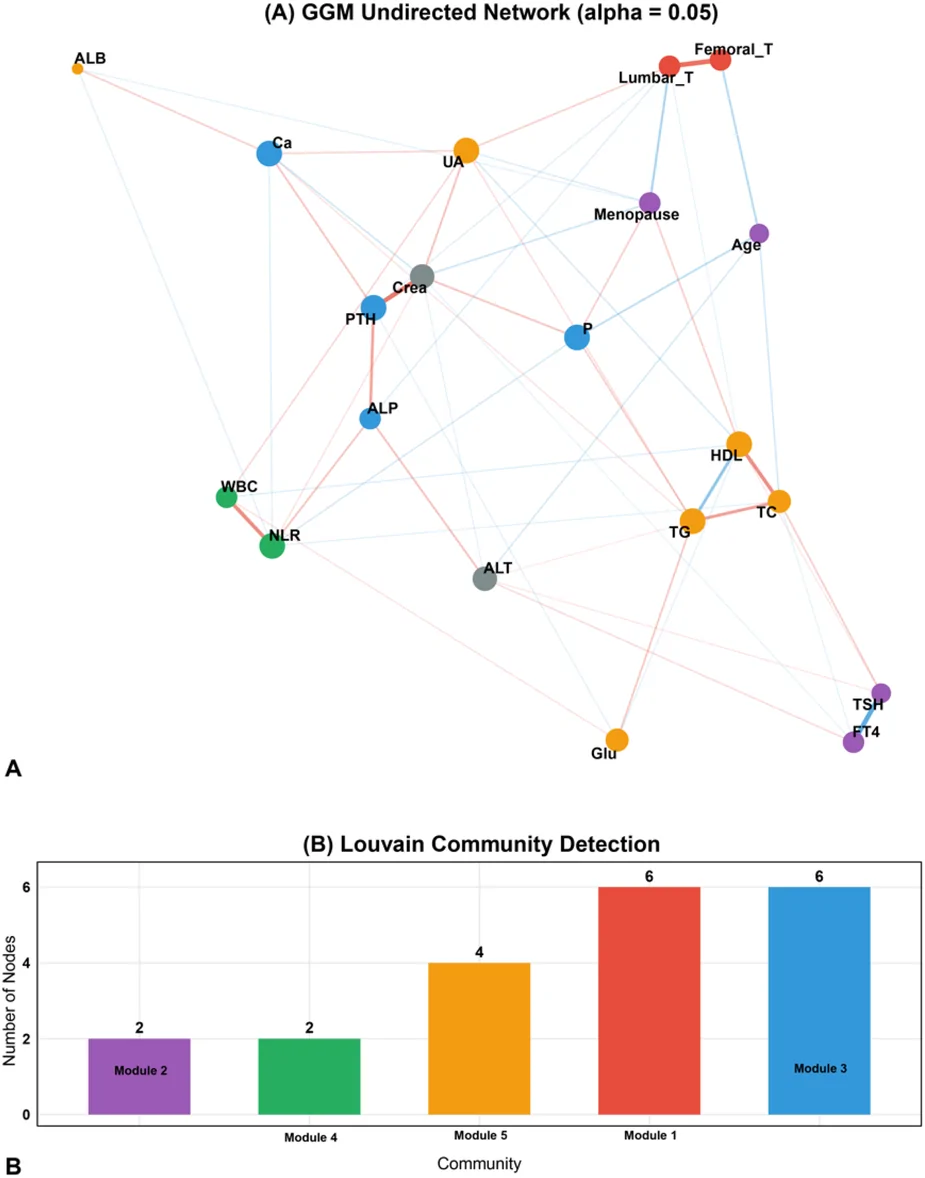
Global system interconnection revealed by Gaussian graphical modeling and community detection. **(A)** Gaussian graphical model (GGM) network topology. Nodes represent 20 variables (19 continuous variables plus menopause status) and are colored according to predefined physiological domains: BMD (red), endocrine function (purple), bone metabolism (blue), immune-inflammatory markers (green), metabolic-nutritional indicators (orange), and hepatic-renal function (gray). Edges represent partial correlations estimated using graphical lasso (alpha = 0.05). Red edges indicate positive partial correlations, whereas blue edges indicate negative partial correlations; edge width is proportional to the absolute correlation coefficient (|r|). The final network contained 71 nonzero edges, corresponding to a network density of 0.37. **(B)** Louvain community detection analysis. Five data-driven communities were identified from the weighted absolute partial-correlation network. BMD nodes clustered with age, menopause status, and phosphorus; TSH, FT4, and ALT formed a separate endocrine-hepatic cluster; Ca, ALP, PTH, ALB, UA, and creatinine formed a cross-domain bone metabolism–nutrition–renal function cluster; NLR and WBC formed an independent inflammatory cluster; and TC, HDL, glucose, and TG formed a lipid-glucose metabolism cluster. These findings indicate substantial overlap between predefined physiological domains.

The final analysis cohort comprised 2,880 individuals with complete lumbar spine and femoral neck T-score records, of whom 1,546 had a postmenopausal status recorded in the electronic medical records. Baseline characteristics of the entire cohort and the postmenopausal subgroup are presented in Supplementary Figure S1 and Table 1. After GGM estimation on the 20-node matrix, 71 non-zero edges were obtained at alpha = 0.05, corresponding to an edge density of 0.37 (71/190) in a 20-node network (Figure 1). The pairs with the strongest partial correlations, in descending order, were TSH–FT4 (r = −0.5312), lumbar spine T-score–femoral neck T-score (r = 0.5304), PTH–creatinine (r = 0.4921), NLR–white blood cells (r = 0.4040), and TC–HDL (r = 0.3946) (Supplementary Table S1).

**Table 1:** Baseline characteristics of the study population.

Variable	Full Cohort (*n* = 2,880)	Menopause = 0 (*n* = 1,334)	Menopause = 1 (*n* = 1,546)
BMD			
Lumbar spine T-score	−0.49 ± 1.87	0.21 ± 1.88	−1.09 ± 1.63
Femoral neck T-score	−1.02 ± 1.22	−0.77 ± 1.23	−1.23 ± 1.17
Demographics			
Age (years)	63.69 ± 9.44	63.19 ± 9.38	64.12 ± 9.48
Bone metabolism biomarkers			
Alkaline phosphatase, ALP (U/L)	65.52 ± 25.39	64.31 ± 26.99	66.56 ± 23.89
Parathyroid hormone, PTH (pg/mL)	40.39 ± 30.15	39.92 ± 30.19	40.80 ± 30.13
Total calcium (mmol/L)	2.25 ± 0.12	2.25 ± 0.11	2.25 ± 0.12
Phosphorus (mmol/L)	1.18 ± 0.18	1.15 ± 0.18	1.21 ± 0.17
Immune-inflammatory markers			
Neutrophil-to-lymphocyte ratio, NLR	2.20 ± 1.64	2.37 ± 1.84	2.05 ± 1.43
White blood cell count (×10^9^/L)	6.27 ± 1.65	6.44 ± 1.61	6.13 ± 1.68
Metabolic-nutritional indicators			
ALB (g/L)	47.73 ± 8.62	48.70 ± 8.66	46.89 ± 8.51
TC (mmol/L)	4.52 ± 1.31	4.36 ± 1.18	4.66 ± 1.40
HDL (mmol/L)	1.30 ± 0.31	1.22 ± 0.28	1.38 ± 0.32
TG (mmol/L)	1.54 ± 1.03	1.53 ± 1.10	1.54 ± 0.98
Glucose (mmol/L)	6.55 ± 2.15	6.63 ± 2.07	6.48 ± 2.21
UA (*μ*mol/L)	317.60 ± 84.69	338.30 ± 84.13	299.74 ± 81.05
Endocrine indicators			
TSH (mIU/L)	3.85 ± 9.06	3.46 ± 7.87	4.18 ± 9.96
Free thyroxine, FT4 (pmol/L)	15.86 ± 4.06	16.01 ± 3.43	15.74 ± 4.54
Hepatic and renal function			
Alanine aminotransferase, ALT (U/L)	22.76 ± 17.55	22.61 ± 14.94	22.89 ± 19.53
Creatinine (μmol/L)	66.85 ± 37.05	74.50 ± 38.05	60.25 ± 34.86

Data are presented as mean ± standard deviation.

“Menopause=1″ indicates participants recorded as postmenopausal in the medical record system.

“Menopause=0″ indicates participants without a documented postmenopausal status and may include premenopausal women, men, or individuals with undocumented menopause status.

Louvain community detection identified five data-driven communities, which did not show a one-to-one correspondence with the *a priori* modules. Community 1 comprised lumbar spine T-score, femoral neck T-score, age, menopause status, and phosphorus; Community 2 comprised TSH, FT4, and ALT; Community 3 comprised Ca, ALP, PTH, ALB, UA, and creatinine; Community 4 consisted of NLR and white blood cells; and Community 5 consisted of TC, HDL, Glu, and TG. This finding suggests that, in the current sample, bone density and age-related nodes formed a tighter conditional dependence structure with certain mineral metabolism indicators, whereas bone metabolism, nutritional, and renal function indicators exhibited cross-system integration (Supplementary Figure S2).

**Figure 2:**
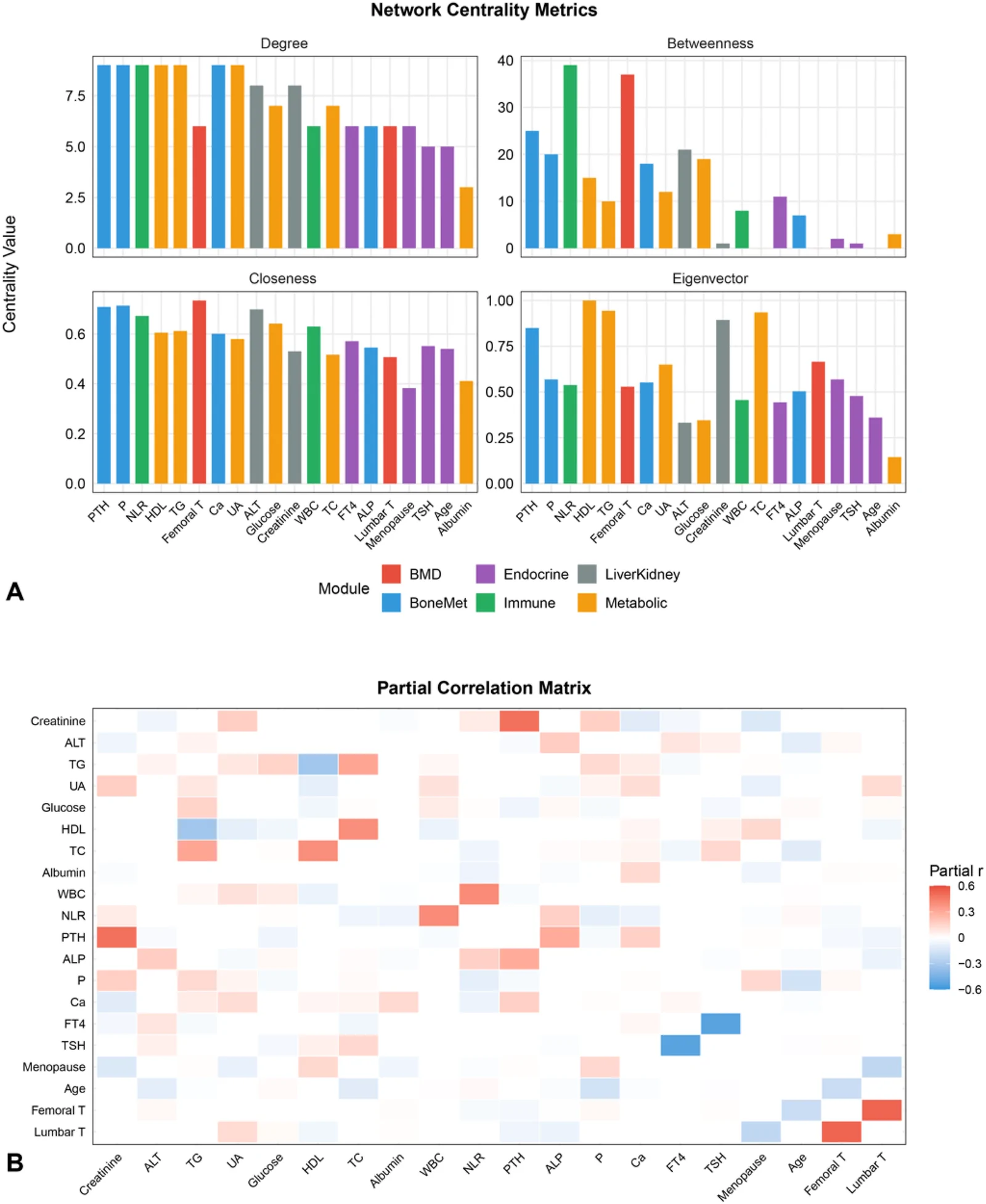
Network centrality metrics and partial correlation matrix. **(A)** Centrality analysis of the Gaussian graphical model network. Degree, betweenness, closeness, and eigenvector centrality were calculated for all 20 nodes. Based on the overall average ranking, PTH, phosphorus, NLR, HDL, and TG exhibited the highest centrality, suggesting prominent hub roles for bone metabolism, inflammatory, and lipid metabolic pathways within the network. **(B)** Partial correlation matrix of all network variables. Colors indicate the direction and magnitude of partial correlations, with red representing positive correlations and blue representing negative correlations. The strongest pairwise associations included TSH–FT4 (*r* = −0.5312), lumbar spine T-score–femoral neck T-score (*r* = 0.5304), PTH–creatinine (*r* = 0.4921), NLR–WBC (*r* = 0.4040), and TC–HDL (*r* = 0.3946).

Based on the average ranking across degree, betweenness, closeness, and eigenvector centrality, the top-ranked nodes were PTH, P, NLR, HDL, and TG (Figure 2). Examining the cross-community bridge ratio, the nodes with the highest bridging capacity were NLR, FT4, WBC, TSH, and TC (Supplementary Figure S3). These results collectively indicate that the network hubs in this sample were not confined to age or calcium alone, but rather reflected intersections among bone metabolism, immune-inflammatory, and lipid metabolism nodes.

### 3.2 Directed network structure under prior constraints

**Figure 3:**
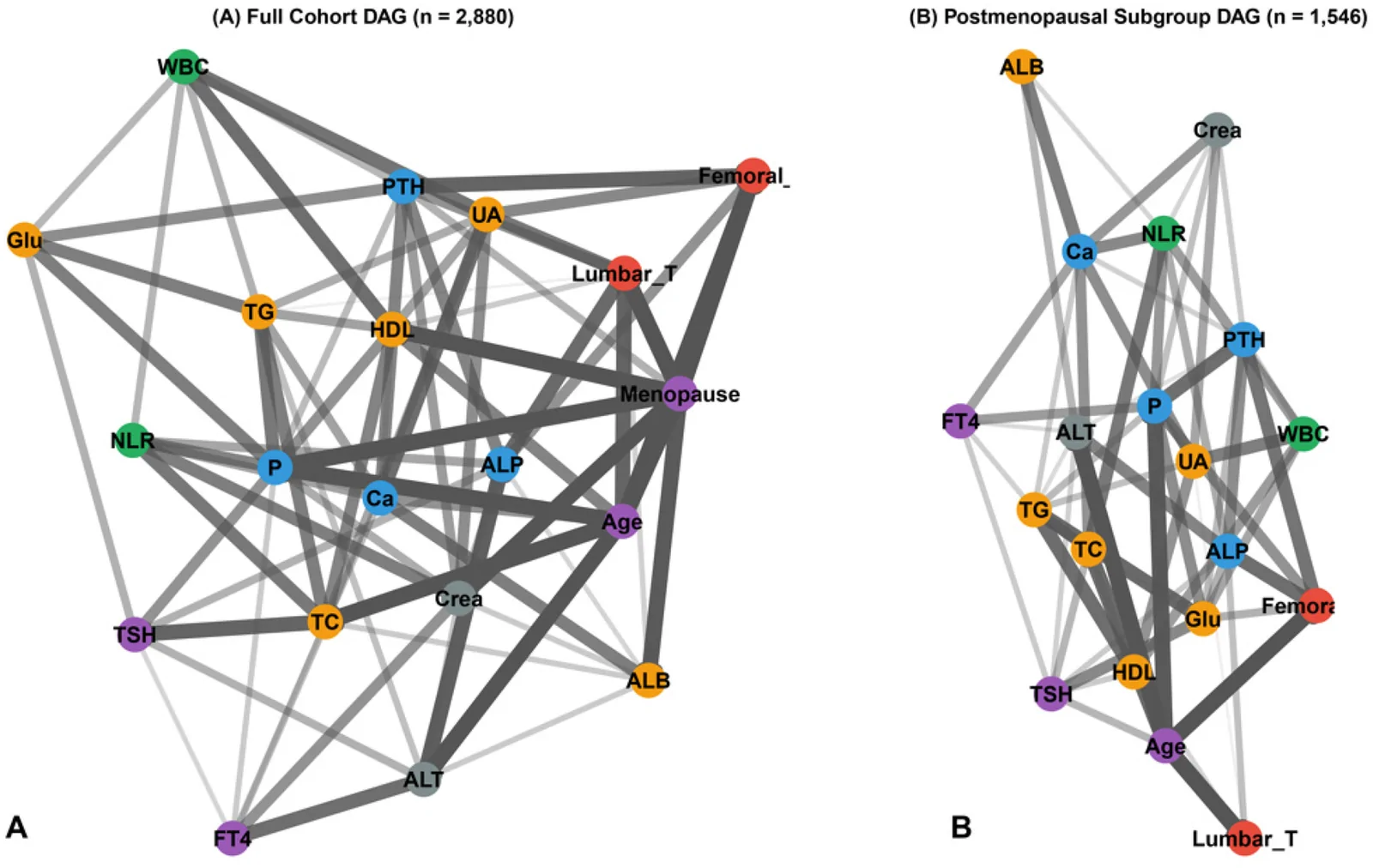
Clinically constrained Bayesian directed acyclic graphs in the full cohort and postmenopausal subgroup. **(A)** Directed acyclic graph (DAG) in the full cohort (*n* = 2,880). A clinically constrained Bayesian network containing 75 directed edges was constructed under prior biological restrictions. Blacklist constraints prohibited any variable from directing toward age, prohibited all variables except age from directing toward menopause status, and prohibited reciprocal directions between lumbar spine and femoral neck T-scores. The whitelist constraint specified Age - Menopause. Major edges directly connected to BMD nodes with bootstrap support ≥50% included Age - Lumbar_T (90.0%), Age - Femoral_T (100.0%), Menopause - Lumbar_T (100.0%), Menopause - Femoral_T (100.0%), PTH - Femoral_T (90.0%), and ALP - Femoral_T (60.0%). **(B)** Directed acyclic graph in the postmenopausal subgroup (*n* = 1,546). The menopause node was excluded from subgroup analysis, and the resulting network retained 63 directed edges. Compared with the full cohort, the support rate for ALP - Femoral_T increased from 60.0% to 83.3%, PTH - Femoral_T remained stable at 80.0%, and a new edge, P - Femoral_T (60.0%), emerged. These findings suggest that, in participants recorded as postmenopausal, the stable directed dependencies between bone metabolism biomarkers and femoral neck BMD were stronger, whereas the direct effects of age persisted.

Building on the undirected association landscape provided by the GGM, we further performed DAG structure learning with prior constraints. A total of 75 directed edges were identified in the whole-cohort DAG, of which 46 had a support rate ≥ 50% across 30 bootstrap resamples (Figure 3, Supplementary Figure S4, Supplementary Table S2). In the final derived output, no edges violating the blacklist were observed, confirming that the constraints successfully prevented prespecified prohibited structures, including variables pointing to age, variables pointing to menopause status, and directed connections between lumbar spine and femoral neck T-scores.

In the whole cohort, edges directly pointing to BMD nodes with support ≥ 50% included Age - Lumbar spine T-score (90.0%), Age - Femoral neck T-score (100.0%), Menopause - Lumbar spine T-score (100.0%), Menopause - Femoral neck T-score (100.0%), PTH - Femoral neck T-score (90.0%), and ALP - Femoral neck T-score (60.0%). This indicates that, under prior constraints, the femoral neck T-score continued to receive stable directed input from bone metabolism biochemical nodes, whereas the stable direct inputs to the lumbar spine T-score were primarily from age and menopause status.

In the postmenopausal subgroup (*n* = 1,546), after removing the Menopause node, the DAG retained 63 directed edges, of which 29 had support ≥ 50% (Supplementary Table S3). Compared with the whole cohort, the support for ALP - Femoral neck T-score increased from 60.0% to 83.3%, PTH - Femoral neck T-score remained at 80.0%, and a new edge, P - Femoral neck T-score (60.0%), emerged. Meanwhile, Age - Lumbar spine T-score and Age - Femoral neck T-score both remained at 100% in the subgroup. Therefore, a more accurate description of the subgroup findings is not that the age effect disappeared, but that the stable directed dependencies involving bone metabolism biomarkers and the femoral neck T-score further increased while the direct effects of age persisted.

### 3.3 Network-informed indirect associations between systemic indicators, bone metabolism biomarkers, and BMD in the whole cohort and postmenopausal subgroup

**Figure 4:**
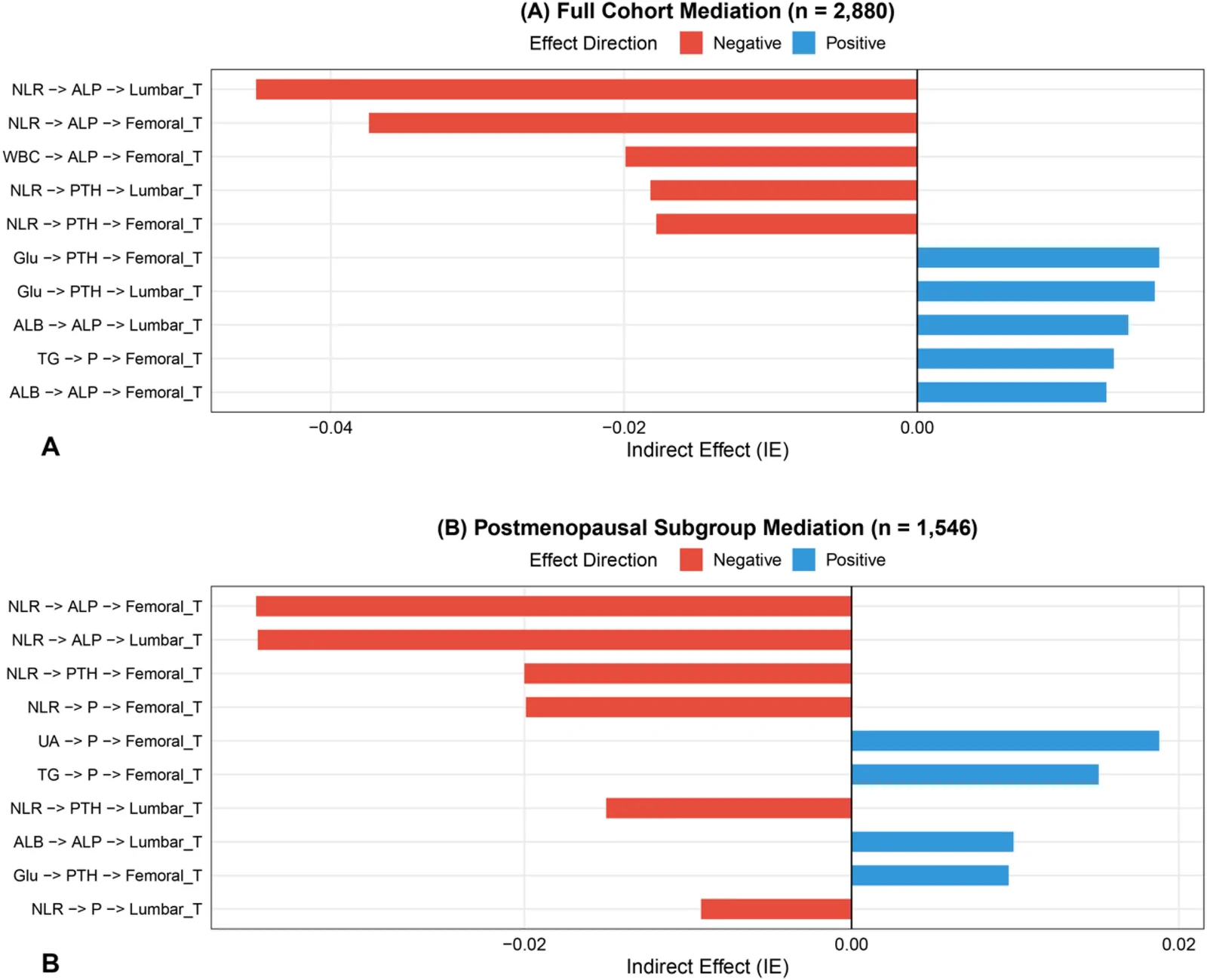
Network-informed bridge mediation analysis linking systemic signals to BMD through bone metabolism biomarkers. **(A)** Bridge mediation analysis in the full cohort (*n* = 2,880). Significant mediation pathways with directionally consistent indirect and direct effects are shown. The strongest negative bridging pathways were NLR - ALP - Lumbar_T (*IE* = −0.0451) and NLR - ALP - Femoral_T (*IE* = −0.0374). Major positive bridging pathways included ALB - ALP - Lumbar_T (*IE* =  + 0.0144) and ALB - ALP - Femoral_T (*IE* =  + 0.0129). **(B)** Bridge mediation analysis in the postmenopausal subgroup (*n* = 1,546). The negative bridging pathways NLR - ALP - Lumbar_T (*IE* = −0.0363) and NLR - ALP - Femoral_T (IE = −0.0364) remained significant in the postmenopausal subgroup. Alternative inflammatory bridging pathways involving NLR - PTH/P - BMD were also retained. The pathway ALB - ALP - Lumbar_T remained significant (*IE* =  + 0.0099); however, the high mediation proportion (*PM* = 94.4%) was primarily attributable to arithmetic inflation caused by a near-zero direct effect rather than a substantial increase in indirect effect magnitude.

Based on the pre-specified bridge mediation framework, we systematically traversed source - bone metabolism biochemical bridge - BMD pathways in the whole cohort and the postmenopausal subgroup, and retained results where the indirect effect had the same sign as the direct effect and the bootstrap 95% confidence interval did not cross zero (Figure 4, Supplementary Table S4). A total of 17 significant pathways were detected in the whole cohort, and 12 in the postmenopausal subgroup.

The most stable signals originated from inflammation-associated ALP bridging patterns. In the whole cohort, the indirect effect of NLR - ALP - Lumbar spine T-score was −0.0451 [95% confidence interval (CI) [−0.0669, −0.0290]; proportion mediated (PM) = 70.5%], and that of NLR - ALP - Femoral neck T-score was −0.0374 [95% CI (−0.0552, −0.0227); PM = 37.9%]. Additionally, NLR - PTH - Lumbar spine T-score, NLR - PTH - Femoral neck T-score, and NLR - P - Femoral neck T-score also reached statistical significance, suggesting that the relationship between inflammatory burden and bone loss is not mediated solely by ALP, but rather involves a set of bridging signals centered on bone metabolism biochemical nodes.

Nutrition-related signals were primarily manifested as ALB - ALP - BMD. In the whole cohort, the indirect effect of ALB - ALP - Lumbar spine T-score was +0.0144 [95% CI (0.0079, 0.0220); PM = 29.6%], and that of ALB - ALP - Femoral neck T-score was +0.0129 [95% CI (0.0070, 0.0200); PM = 22.2%]. Based on the coefficient directions, higher ALB was associated with lower ALP, which in turn co-occurred with higher BMD.

In the postmenopausal subgroup, the inflammation-associated ALP bridging signals persisted: the indirect effect of NLR - ALP - Lumbar spine T-score was −0.0363 (PM = 48.6%), and that of NLR - ALP - Femoral neck T-score was −0.0364 (PM = 45.0%); NLR - PTH - Lumbar spine T-score, NLR - PTH - Femoral neck T-score, NLR - P - Lumbar spine T-score, and NLR - P - Femoral neck T-score also reached statistical significance. This indicates that after eliminating the intergroup variation driven by menopause status, the inflammation-related bridging signals did not disappear, but were preserved in the form of multiple bone metabolism biochemical pathways.

In the postmenopausal subgroup, the ALB–ALP–lumbar spine BMD indirect association remained significant in the primary analysis, with an indirect effect of +0.0099 [95% CI (0.0027, 0.0185); PM = 94.4%]. It should be objectively noted that this extremely high mediation proportion was largely driven by arithmetic inflation resulting from a direct effect near zero (DE = + 0.0006), rather than a substantial increase in the absolute magnitude of the indirect effect. Therefore, it should not be interpreted as ALB acting almost entirely through ALP.

Metabolism-related bridging signals were more complex. In the whole cohort, Glu - PTH - Lumbar spine T-score and Glu - PTH - Femoral neck T-score showed positive indirect effects; TG - P - Femoral neck T-score and UA - P - Femoral neck T-score were also positive. In the postmenopausal subgroup, the positive indirect effects of Glu - PTH, TG - P, and UA - P persisted. Considering that BMI was not included in the model and that DXA is sensitive to soft tissue and degenerative changes, these signals, where metabolic indicators co-occur with higher BMD, should be understood as a mixed reflection of body weight/adiposity surrogate effects and measurement artifacts, rather than being directly interpreted as metabolically driven bone protection.

### 3.4 Sensitivity and subgroup analyses

Adjustment for ALT, GGT, and total bilirubin produced little to modest attenuation of the NLR–ALP–BMD indirect associations, which remained statistically significant in both cohorts (full cohort lumbar spine: IE −0.0262, 95% CI −0.0430 to −0.0130; femoral neck: IE −0.0290, 95% CI −0.0435 to −0.0164; postmenopausal subgroup: lumbar spine IE −0.0255, 95% CI −0.0453 to −0.0117; femoral neck IE −0.0273, 95% CI −0.0437 to −0.0138; Supplementary Table S5; Supplementary Figure S5). These findings reduce the possibility that the associations were explained by measured hepatic abnormalities.

The negative NLR–ALP–BMD indirect associations remained statistically significant across all prespecified subgroup definitions (ALP ≤ 125 U/L, ALT < 40 U/L, both conditions, ALP 45–125 U/L, and ALT < 50 U/L), in both BMD sites and both cohorts (Supplementary Table S6); the ALB–ALP–BMD associations were not consistently observed in the ALP-normal subgroups or in the postmenopausal subgroups. The core NLR–ALP–BMD indirect associations also remained significant in the same-sample baseline analysis restricted to participants with complete hepatic-marker data.

## 4 Discussion

This study systematically characterized the conditional and directed dependency structures between BMD and endocrine, immune-inflammatory, metabolic-nutritional, and hepatic-renal indicators using a three-tier framework of undirected topology, prior-constrained directed network, and bridge mediation. The results support the hypothesis that osteoporosis is a network-level disorder involving coordinated instability across multiple physiological systems. This study incorporated biological plausibility into the structure learning process by blacklisting physiologically implausible edges, thereby restricting the directed acyclic graph to biologically admissible pathways and improving the interpretability of the estimated directed dependency structure.

The most central finding was that the NLR-ALP-BMD pathway maintained a consistent negative indirect effect in both the whole cohort and the postmenopausal subgroup. This negative indirect association remained statistically significant after adjustment for ALT, GGT, and total bilirubin and across the normal-ALP and normal-ALT subgroup definitions. This observation aligns with growing epidemiological evidence linking elevated NLR to osteoporosis and fracture risk (13, 14). The present study extends these associations by suggesting that inflammatory burden may be indirectly associated with BMD through bone metabolism biomarkers rather than through a direct pathway. Recent experimental studies further support this interpretation, showing that neutrophil extracellular traps (NETs) impair osteoblast differentiation through NCF2-dependent signaling (15). Thus, elevated NLR may reflect not only increased neutrophil proportion but also a state of NETs-mediated suppression of bone formation. ALP, although traditionally regarded as a marker of osteoblast activity (16), likely reflects increased bone remodeling activity in osteoporosis, where bone resorption exceeds formation (17). The negative indirect effect observed in this study is therefore biologically coherent: higher inflammatory burden co-occurs with higher total ALP and lower BMD. Importantly, the inflammatory signal was not restricted to a single pathway. Both NLR-PTH-BMD and NLR-P-BMD were also significant, suggesting that inflammation–bone interactions operate through multiple parallel biochemical bridges rather than a single mediator. This pattern is highly consistent with the concept of immunoporosis, in which immune cells regulate bone remodeling through interconnected inflammatory and osteoclastogenic pathways (3, 18). In the postmenopausal subgroup, the NLR-ALP-BMD signal persisted and became more focused, supporting the notion that the inflammatory association remained observable in the postmenopausal subgroup (19).

The ALB-ALP-BMD pathway represented a weaker, exploratory signal that attenuated after adjustment for hepatic markers and was not consistently observed in the postmenopausal or normal-ALP subgroups; a partial hepatic/systemic contribution cannot be excluded. Better nutritional status co-occurred with lower ALP and higher BMD, suggesting that malnutrition may contribute to skeletal fragility partly through increased bone remodeling activity (20, 21). Accordingly, this pathway should be interpreted as a nutritional–bone metabolism balance signal rather than evidence of a direct anabolic role of albumin in bone metabolism. Several metabolism-related pathways (Glu-PTH-BMD, TG-P-BMD, and UA-P-BMD) showed positive indirect effects, indicating that higher metabolic indices co-occurred with higher BMD. However, these findings require careful interpretation. First, BMI was unavailable in more than half of participants, and the missingness pattern suggested systematic collection bias. Because glucose, triglycerides, and UA strongly correlate with adiposity, these variables likely functioned as partial surrogates for unmeasured obesity information (22, 23). Second, DXA-derived areal BMD is susceptible to measurement artifacts caused by soft tissue thickness, spinal degeneration, osteophytes, and vascular calcification (24). Therefore, the observed positive metabolic associations should not be interpreted as evidence that hyperglycemia or dyslipidemia protect bone, but rather as a combined reflection of adiposity-related mechanical loading and DXA measurement bias.

These findings have practical implications. NLR may help identify patients in whom elevated total ALP co-occurs with systemic inflammatory burden, while ALB should be interpreted alongside BMD as a marker of nutritional status. Positive associations between metabolic indicators and BMD also caution against relying solely on areal DXA in obese or diabetic populations. However, this study has several limitations. The cross-sectional design precludes definitive causal inference despite the use of prior constraints. The systematic absence of BMI and CRP may have biased effect estimates, and the inclusion of a binary menopause variable in the Gaussian graphical model deviates from strict multivariate normality assumptions. In addition, the single-center retrospective design limits generalizability to broader community-based or multi-ethnic populations. Prospective studies are needed to validate the temporal sequence of the core NLR-ALP-BMD pathway and determine whether anti-inflammatory interventions can mitigate bone loss. Bone-specific ALP, P1NP, and CTX-I were unavailable in the hospital information system. Therefore, total ALP was used only as a non-specific surrogate of bone remodeling activity, and its tissue origin could not be determined.

## 5 Conclusion

In summary, by integrating undirected topology, prior-constrained directed network learning, and bridge mediation, this study provides evidence that systemic inflammation is associated with BMD through bone-metabolism biochemical bridges, with NLR-ALP-BMD emerging as the most robust negative signal; a weaker nutritional signal (ALB-ALP-BMD) was observed in the whole cohort but was not consistently observed in the postmenopausal subgroup after adjustment for hepatic markers. The explicit incorporation of clinical priors improves the interpretability of the estimated directed dependency structure and may provide a practical framework for future bone–immune–metabolic network studies. The cross-sectional design precludes causal inference.

## Data Availability

The original contributions presented in the study are included in the article/Supplementary Material, further inquiries can be directed to the corresponding authors.
