# Supplementary material for: Clinically constrained Bayesian network analysis of immune–metabolic factors associated with bone mineral density in a real-world orthopedic cohort
Source: Front Surg. 2026 Aug 19;13:1934871. doi: 10.3389/fsurg.2026.1934871 (PMC13533923; doi:10.3389/fsurg.2026.1934871)
Supplement: Supplementary file 1 [file Table1.docx]

**Supplementary Figure 1. Baseline characteristics of the full cohort and postmenopausal subgroup.**

Representative distributions of 12 variables in the full cohort and the postmenopausal subgroup are shown. Each variable is displayed using violin plots overlaid with boxplots to visualize differences in the distributions of bone mineral density, inflammatory markers, bone metabolism indicators, and metabolic-nutritional variables between the two populations.

**Supplementary Figure 2. Heatmap of inter-module partial correlation strength.**

Heatmap showing the mean absolute partial correlation strengths among six predefined physiological modules. Diagonal elements represent the average within-module partial correlation strength, whereas off-diagonal elements represent the average coupling strength between modules. The analysis provides a system-level overview of conditional dependency relationships across physiological domains.

**Supplementary Figure 3. Bridge node strength across data-driven network communities.**

Bridge ratios of all 20 nodes across data-driven communities are shown, calculated as the number of inter-community edges divided by the total number of edges for each node. NLR exhibited the highest bridge ratio, followed by FT4, WBC, TSH, and TC, suggesting prominent cross-module connectivity roles for inflammatory and thyroid-related pathways within the network.

**Supplementary Figure 4. Bootstrap stability ranking of directed edges in the full-cohort DAG.**

Bootstrap support rates for directed edges in the full-cohort directed acyclic graph (DAG) are ranked in descending order. Only edges with bootstrap support ≥50% are shown, and the dashed line indicates the 50% threshold. Red points represent edges directly connected to bone mineral density nodes, whereas gray points represent all other edges. The final results were derived from 30 bootstrap resampling iterations.

**Supplementary Figure 5. Sensitivity analysis of core bridge-mediation pathways with adjustment for hepatic markers.**

*Forest plots showing the indirect effects (IE) with 95% bootstrap confidence intervals (CI) for the four core bridge-mediation pathways (NLR-ALP-BMD and ALB-ALP-BMD for the lumbar spine and femoral neck) in the full cohort (A) and the postmenopausal subgroup (B). Three nested adjustment models were applied on the identical sample (n = 2,178 in the full cohort; n = 1,251 in the postmenopausal subgroup): the base model (blue; covariates: age and menopause status in the full cohort, age alone in the postmenopausal subgroup), the base model additionally adjusted for ALT (orange), and the base model additionally adjusted for ALT, GGT, and total bilirubin (red). The dashed vertical line indicates the null effect (IE = 0). The NLR-ALP-BMD pathways remained statistically significant in all models, whereas the ALB-ALP-BMD pathways were attenuated after adjustment and were no longer significant in the postmenopausal subgroup.*

**Supplementary Table 1. Top 40 GGM Edges Ranked by Absolute Partial Correlation**

| **Rank** | **Node A** | **Node B** | **Partial Correlation (r)** | **Direction** |
| --- | --- | --- | --- | --- |
| 1 | TSH | FT4 | −0.5312 | Negative |
| 2 | Lumbar_T | Femoral_T | 0.5304 | Positive |
| 3 | PTH | Creatinine | 0.4921 | Positive |
| 4 | NLR | WBC | 0.4040 | Positive |
| 5 | TC | HDL-C | 0.3946 | Positive |
| 6 | HDL-C | TG | −0.3216 | Negative |
| 7 | TC | TG | 0.3160 | Positive |
| 8 | ALP | PTH | 0.2923 | Positive |
| 9 | Lumbar_T | Menopause | −0.2230 | Negative |
| 10 | Femoral_T | Age | −0.1885 | Negative |
| 11 | ALP | ALT | 0.1752 | Positive |
| 12 | UA | Creatinine | 0.1728 | Positive |
| 13 | P | Creatinine | 0.1661 | Positive |
| 14 | ALP | NLR | 0.1647 | Positive |
| 15 | Ca | PTH | 0.1643 | Positive |
| 16 | Age | P | −0.1635 | Negative |
| 17 | Glucose | TG | 0.1547 | Positive |
| 18 | TSH | TC | 0.1364 | Positive |
| 19 | Menopause | P | 0.1350 | Positive |
| 20 | Menopause | HDL-C | 0.1345 | Positive |
| 21 | P | TG | 0.1301 | Positive |
| 22 | Ca | ALB | 0.1296 | Positive |
| 23 | Lumbar_T | UA | 0.1277 | Positive |
| 24 | Menopause | Creatinine | −0.1250 | Negative |
| 25 | Ca | UA | 0.1185 | Positive |
| 26 | Ca | Creatinine | −0.1084 | Negative |
| 27 | Age | TC | −0.1065 | Negative |
| 28 | WBC | UA | 0.1045 | Positive |
| 29 | Age | ALT | −0.0950 | Negative |
| 30 | FT4 | ALT | 0.0925 | Positive |
| 31 | HDL-C | UA | −0.0905 | Negative |
| 32 | P | NLR | −0.0904 | Negative |
| 33 | UA | TG | 0.0859 | Positive |
| 34 | Menopause | UA | −0.0768 | Negative |
| 35 | NLR | Creatinine | 0.0699 | Positive |
| 36 | Lumbar_T | ALP | −0.0688 | Negative |
| 37 | WBC | HDL-C | −0.0684 | Negative |
| 38 | Ca | TG | 0.0661 | Positive |
| 39 | WBC | Glucose | 0.0655 | Positive |
| 40 | Ca | NLR | −0.0654 | Negative |

*The Gaussian graphical model (GGM) was estimated using graphical lasso (alpha = 0.05). The final 20-node network contained 71 nonzero edges; the present table lists the top 40 edges ranked by absolute partial correlation magnitude.*

**Supplementary Table 2. Full-Cohort DAG Edges with Bootstrap Support ≥ 50% (n = 2,880)**

| **Source Node** | **Target Node** | **Bootstrap Support (%)** |
| --- | --- | --- |
| Age | ALT | 100.0 |
| Age | Femoral_T | 100.0 |
| Age | Menopause | 100.0 |
| Age | P | 100.0 |
| Age | TC | 100.0 |
| Menopause | Creatinine | 100.0 |
| Menopause | Femoral_T | 100.0 |
| Menopause | HDL | 100.0 |
| Menopause | Lumbar_T | 100.0 |
| Menopause | P | 100.0 |
| ALT | ALP | 93.3 |
| Age | Lumbar_T | 90.0 |
| PTH | Femoral_T | 90.0 |
| TC | TSH | 90.0 |
| Lumbar_T | ALP | 86.7 |
| ALT | FT4 | 83.3 |
| Ca | UA | 83.3 |
| HDL | WBC | 83.3 |
| UA | WBC | 80.0 |
| Lumbar_T | UA | 76.7 |
| P | TG | 76.7 |
| TC | NLR | 76.7 |
| Ca | ALB | 73.3 |
| Creatinine | NLR | 73.3 |
| P | Glucose | 73.3 |
| TG | Glucose | 73.3 |
| TG | TC | 73.3 |
| Age | HDL | 70.0 |
| Ca | PTH | 70.0 |
| HDL | TC | 70.0 |
| P | NLR | 70.0 |
| PTH | ALP | 70.0 |
| UA | Femoral_T | 66.7 |
| Creatinine | UA | 63.3 |
| PTH | Glucose | 63.3 |
| ALP | Femoral_T | 60.0 |
| Age | Ca | 60.0 |
| Creatinine | FT4 | 60.0 |
| HDL | TSH | 60.0 |
| Lumbar_T | PTH | 60.0 |
| Creatinine | PTH | 53.3 |
| Ca | NLR | 50.0 |
| Ca | TG | 50.0 |
| HDL | UA | 50.0 |
| TG | HDL | 50.0 |

*The full-cohort directed acyclic graph (DAG) contained 75 edges, of which 46 exhibited bootstrap support ≥50%. No edges violating blacklist constraints were observed in the final exported network.*

**Supplementary Table 3. Postmenopausal-Subgroup DAG Edges with Bootstrap Support ≥ 50% (n = 1,546)**

| **Source Node** | **Target Node** | **Bootstrap Support (%)** |
| --- | --- | --- |
| Age | ALT | 100.0 |
| Age | Femoral_T | 100.0 |
| Age | Lumbar_T | 100.0 |
| Age | P | 96.7 |
| Age | TC | 86.7 |
| TG | Glucose | 86.7 |
| ALP | Femoral_T | 83.3 |
| TG | HDL | 83.3 |
| P | PTH | 83.3 |
| PTH | Femoral_T | 80.0 |
| TC | NLR | 76.7 |
| ALB | Ca | 73.3 |
| UA | WBC | 73.3 |
| ALT | ALP | 70.0 |
| Ca | NLR | 66.7 |
| TSH | Glucose | 66.7 |
| ALP | PTH | 63.3 |
| Ca | UA | 63.3 |
| P | Glucose | 63.3 |
| HDL | UA | 60.0 |
| P | Femoral_T | 60.0 |
| TC | Ca | 60.0 |
| TG | TC | 60.0 |
| Ca | Creatinine | 56.7 |
| P | NLR | 56.7 |
| FT4 | P | 53.3 |
| HDL | WBC | 53.3 |
| NLR | WBC | 53.3 |
| FT4 | Ca | 50.0 |

*After removal of the menopause node, the postmenopausal-subgroup DAG retained 63 directed edges, of which 29 showed bootstrap support ≥50%. In this subgroup, ALP → Femoral_T, PTH → Femoral_T, and P → Femoral_T jointly constituted the major direct biochemical inputs to femoral neck bone mineral density.*

**Supplementary Table 4. Significant Bridge-Mediation Pathways in the Full Cohort and the Postmenopausal Subgroup**

A. Full Cohort (17 Significant Pathways)

| **Pathway** | **IE** | **95% CI (IE)** | **PM (%)** |
| --- | --- | --- | --- |
| NLR → ALP → Lumbar_T | −0.0451 | [−0.0669, −0.0290] | 70.5 |
| NLR → ALP → Femoral_T | −0.0374 | [−0.0552, −0.0227] | 37.9 |
| NLR → PTH → Lumbar_T | −0.0182 | [−0.0330, −0.0086] | 28.5 |
| NLR → PTH → Femoral_T | −0.0178 | [−0.0317, −0.0083] | 18.0 |
| NLR → P → Femoral_T | −0.0072 | [−0.0138, −0.0026] | 7.3 |
| ALB → ALP → Lumbar_T | +0.0144 | [0.0079, 0.0220] | 29.6 |
| ALB → ALP → Femoral_T | +0.0129 | [0.0070, 0.0200] | 22.2 |
| TG → P → Femoral_T | +0.0134 | [0.0061, 0.0229] | 32.9 |
| UA → P → Femoral_T | +0.0112 | [0.0045, 0.0203] | 14.2 |
| UA → ALP → Lumbar_T | +0.0073 | [0.0008, 0.0148] | 4.0 |
| UA → ALP → Femoral_T | +0.0067 | [0.0010, 0.0129] | 8.5 |
| HDL → P → Femoral_T | −0.0038 | [−0.0083, −0.0009] | 5.9 |
| TC → ALP → Lumbar_T | −0.0071 | [−0.0138, −0.0012] | 78.7 |
| TC → ALP → Femoral_T | −0.0064 | [−0.0122, −0.0016] | 92.9 |
| Glucose → PTH → Lumbar_T | +0.0162 | [0.0099, 0.0230] | 18.4 |
| Glucose → PTH → Femoral_T | +0.0165 | [0.0105, 0.0240] | 29.4 |
| WBC → ALP → Femoral_T | −0.0199 | [−0.0293, −0.0107] | 55.5 |

B. Postmenopausal Subgroup (12 Significant Pathways)

| **Pathway** | **IE** | **95% CI (IE)** | **PM (%)** |
| --- | --- | --- | --- |
| NLR → ALP → Lumbar_T | −0.0363 | [−0.0584, −0.0215] | 48.6 |
| NLR → ALP → Femoral_T | −0.0364 | [−0.0602, −0.0196] | 45.0 |
| NLR → PTH → Lumbar_T | −0.0150 | [−0.0260, −0.0082] | 20.0 |
| NLR → PTH → Femoral_T | −0.0200 | [−0.0337, −0.0113] | 24.7 |
| NLR → P → Lumbar_T | −0.0092 | [−0.0179, −0.0001] | 12.3 |
| NLR → P → Femoral_T | −0.0199 | [−0.0327, −0.0094] | 24.6 |
| ALB → ALP → Lumbar_T | +0.0099 | [0.0027, 0.0185] | 94.4* |
| TG → P → Femoral_T | +0.0151 | [0.0052, 0.0266] | 35.5 |
| UA → P → Femoral_T | +0.0188 | [0.0091, 0.0309] | 55.2 |
| HDL → P → Femoral_T | −0.0066 | [−0.0134, −0.0010] | 11.5 |
| Glucose → PTH → Lumbar_T | +0.0071 | [0.0004, 0.0146] | 10.7 |
| Glucose → PTH → Femoral_T | +0.0096 | [0.0019, 0.0200] | 18.7 |

** The extremely high PM value observed for ALB → ALP → Lumbar_T primarily reflects a near-zero direct effect rather than an unusually large indirect effect magnitude.*

**Supplementary Table 5. Sensitivity analysis of the core bridge-mediation pathways with adjustment for hepatic markers.**

| Cohort | Pathway | Model | n | IE | 95% CI | DE | TE | PM (%) |
| --- | --- | --- | --- | --- | --- | --- | --- | --- |
| Full cohort | ALB → ALP → Femoral neck | M1 (imputed cohort) | 2880 | 0.0129 | [0.0065, 0.0200] | 0.0451 | 0.0580 | 22.2 |
| Full cohort | ALB → ALP → Femoral neck | M1 (same-sample baseline) | 2178 | 0.0092 | [0.0035, 0.0158] | 0.0403 | 0.0495 | 18.5 |
| Full cohort | ALB → ALP → Femoral neck | M2 (+ALT) | 2178 | 0.0090 | [0.0029, 0.0153] | 0.0429 | 0.0519 | 17.4 |
| Full cohort | ALB → ALP → Femoral neck | M3 (+ALT+GGT+TBIL) | 2178 | 0.0066 | [0.0008, 0.0120] | 0.0436 | 0.0502 | 13.2 |
| Full cohort | ALB → ALP → Lumbar spine | M1 (imputed cohort) | 2880 | 0.0144 | [0.0078, 0.0206] | 0.0343 | 0.0487 | 29.6 |
| Full cohort | ALB → ALP → Lumbar spine | M1 (same-sample baseline) | 2178 | 0.0088 | [0.0037, 0.0141] | 0.0218 | 0.0306 | 28.8 |
| Full cohort | ALB → ALP → Lumbar spine | M2 (+ALT) | 2178 | 0.0084 | [0.0032, 0.0143] | 0.0238 | 0.0322 | 26.2 |
| Full cohort | ALB → ALP → Lumbar spine | M3 (+ALT+GGT+TBIL) | 2178 | 0.0058 | [0.0013, 0.0101] | 0.0253 | 0.0311 | 18.6 |
| Full cohort | NLR → ALP → Femoral neck | M1 (imputed cohort) | 2880 | -0.0374 | [-0.0564, -0.0244] | -0.0613 | -0.0986 | 37.9 |
| Full cohort | NLR → ALP → Femoral neck | M1 (same-sample baseline) | 2178 | -0.0290 | [-0.0453, -0.0146] | -0.0740 | -0.1030 | 28.1 |
| Full cohort | NLR → ALP → Femoral neck | M2 (+ALT) | 2178 | -0.0335 | [-0.0527, -0.0183] | -0.0697 | -0.1032 | 32.5 |
| Full cohort | NLR → ALP → Femoral neck | M3 (+ALT+GGT+TBIL) | 2178 | -0.0290 | [-0.0435, -0.0164] | -0.0733 | -0.1023 | 28.4 |
| Full cohort | NLR → ALP → Lumbar spine | M1 (imputed cohort) | 2880 | -0.0451 | [-0.0669, -0.0281] | -0.0189 | -0.0640 | 70.5 |
| Full cohort | NLR → ALP → Lumbar spine | M1 (same-sample baseline) | 2178 | -0.0291 | [-0.0486, -0.0150] | -0.0478 | -0.0769 | 37.9 |
| Full cohort | NLR → ALP → Lumbar spine | M2 (+ALT) | 2178 | -0.0327 | [-0.0520, -0.0169] | -0.0443 | -0.0770 | 42.4 |
| Full cohort | NLR → ALP → Lumbar spine | M3 (+ALT+GGT+TBIL) | 2178 | -0.0262 | [-0.0430, -0.0130] | -0.0429 | -0.0691 | 37.9 |
| Postmenopausal | ALB → ALP → Femoral neck | M1 (imputed cohort) | 1546 | 0.0100 | [0.0018, 0.0197] | -0.0006 | 0.0094 | 105.8 |
| Postmenopausal | ALB → ALP → Femoral neck | M1 (same-sample baseline) | 1251 | 0.0078 | [0.0003, 0.0166] | 0.0084 | 0.0162 | 48.2 |
| Postmenopausal | ALB → ALP → Femoral neck | M2 (+ALT) | 1251 | 0.0058 | [-0.0018, 0.0148] | 0.0143 | 0.0201 | 28.7 |
| Postmenopausal | ALB → ALP → Femoral neck | M3 (+ALT+GGT+TBIL) | 1251 | 0.0028 | [-0.0049, 0.0110] | 0.0137 | 0.0166 | 17.1 |
| Postmenopausal | ALB → ALP → Lumbar spine | M1 (imputed cohort) | 1546 | 0.0099 | [0.0022, 0.0172] | 0.0006 | 0.0104 | 94.4 |
| Postmenopausal | ALB → ALP → Lumbar spine | M1 (same-sample baseline) | 1251 | 0.0074 | [0.0003, 0.0147] | -0.0034 | 0.0040 | 183.9 |
| Postmenopausal | ALB → ALP → Lumbar spine | M2 (+ALT) | 1251 | 0.0056 | [-0.0027, 0.0141] | 0.0036 | 0.0092 | 60.8 |
| Postmenopausal | ALB → ALP → Lumbar spine | M3 (+ALT+GGT+TBIL) | 1251 | 0.0027 | [-0.0052, 0.0103] | 0.0072 | 0.0099 | 27.0 |
| Postmenopausal | NLR → ALP → Femoral neck | M1 (imputed cohort) | 1546 | -0.0364 | [-0.0584, -0.0204] | -0.0445 | -0.0809 | 45.0 |
| Postmenopausal | NLR → ALP → Femoral neck | M1 (same-sample baseline) | 1251 | -0.0340 | [-0.0603, -0.0158] | -0.0600 | -0.0940 | 36.2 |
| Postmenopausal | NLR → ALP → Femoral neck | M2 (+ALT) | 1251 | -0.0391 | [-0.0646, -0.0222] | -0.0557 | -0.0948 | 41.2 |
| Postmenopausal | NLR → ALP → Femoral neck | M3 (+ALT+GGT+TBIL) | 1251 | -0.0273 | [-0.0437, -0.0138] | -0.0608 | -0.0880 | 31.0 |
| Postmenopausal | NLR → ALP → Lumbar spine | M1 (imputed cohort) | 1546 | -0.0363 | [-0.0596, -0.0219] | -0.0385 | -0.0748 | 48.6 |
| Postmenopausal | NLR → ALP → Lumbar spine | M1 (same-sample baseline) | 1251 | -0.0313 | [-0.0529, -0.0145] | -0.0643 | -0.0956 | 32.8 |
| Postmenopausal | NLR → ALP → Lumbar spine | M2 (+ALT) | 1251 | -0.0376 | [-0.0626, -0.0199] | -0.0592 | -0.0968 | 38.8 |
| Postmenopausal | NLR → ALP → Lumbar spine | M3 (+ALT+GGT+TBIL) | 1251 | -0.0255 | [-0.0453, -0.0117] | -0.0550 | -0.0806 | 31.7 |

*The indirect effect (IE), 95% bootstrap confidence interval (CI), direct effect (DE), total effect (TE), and proportion mediated (PM) are shown before and after adjustment. Model M1 (imputed cohort) reproduces the primary analysis on the full imputed cohort (n = 2,880 / n = 1,546); models M1 (same-sample baseline), M2, and M3 were run on the identical same-sample baseline sample with raw ALT, GGT, and total bilirubin (TBIL) values available (n = 2,178 in the full cohort; n = 1,251 in the postmenopausal subgroup). Covariates: age and menopause status (full cohort) or age alone (postmenopausal subgroup). Statistical significance was defined as a bootstrap 95% CI excluding zero.*

**Supplementary Table 6. Subgroup analyses of the core bridge-mediation pathways restricted to participants with ALP within the normal range and/or without elevated ALT.**

| Cohort | Subgroup | Pathway | Model | n | IE | 95% CI | PM (%) |
| --- | --- | --- | --- | --- | --- | --- | --- |
| Full cohort | ALP ≤ 125 U/L | ALB → ALP → Femoral neck | M1 (same-sample baseline) | 2127 | 0.0050 | [0.0004, 0.0102] | 10.7 |
| Full cohort | ALP ≤ 125 U/L | ALB → ALP → Femoral neck | M3 (+ALT+GGT+TBIL) | 2127 | 0.0040 | [-0.0013, 0.0097] | 8.2 |
| Full cohort | ALP ≤ 125 U/L | ALB → ALP → Lumbar spine | M1 (same-sample baseline) | 2127 | 0.0047 | [0.0001, 0.0104] | 14.5 |
| Full cohort | ALP ≤ 125 U/L | ALB → ALP → Lumbar spine | M3 (+ALT+GGT+TBIL) | 2127 | 0.0036 | [-0.0014, 0.0089] | 10.2 |
| Full cohort | ALP ≤ 125 U/L | NLR → ALP → Femoral neck | M1 (same-sample baseline) | 2127 | -0.0161 | [-0.0264, -0.0085] | 15.0 |
| Full cohort | ALP ≤ 125 U/L | NLR → ALP → Femoral neck | M3 (+ALT+GGT+TBIL) | 2127 | -0.0181 | [-0.0292, -0.0098] | 17.0 |
| Full cohort | ALP ≤ 125 U/L | NLR → ALP → Lumbar spine | M1 (same-sample baseline) | 2127 | -0.0154 | [-0.0246, -0.0081] | 21.1 |
| Full cohort | ALP ≤ 125 U/L | NLR → ALP → Lumbar spine | M3 (+ALT+GGT+TBIL) | 2127 | -0.0169 | [-0.0289, -0.0093] | 24.8 |
| Full cohort | ALT < 40 U/L | ALB → ALP → Femoral neck | M1 (same-sample baseline) | 1966 | 0.0086 | [0.0030, 0.0146] | 19.4 |
| Full cohort | ALT < 40 U/L | ALB → ALP → Femoral neck | M3 (+ALT+GGT+TBIL) | 1966 | 0.0071 | [0.0010, 0.0139] | 16.5 |
| Full cohort | ALT < 40 U/L | ALB → ALP → Lumbar spine | M1 (same-sample baseline) | 1966 | 0.0074 | [0.0017, 0.0133] | 23.5 |
| Full cohort | ALT < 40 U/L | ALB → ALP → Lumbar spine | M3 (+ALT+GGT+TBIL) | 1966 | 0.0058 | [0.0009, 0.0107] | 18.5 |
| Full cohort | ALT < 40 U/L | NLR → ALP → Femoral neck | M1 (same-sample baseline) | 1966 | -0.0314 | [-0.0491, -0.0165] | 32.1 |
| Full cohort | ALT < 40 U/L | NLR → ALP → Femoral neck | M3 (+ALT+GGT+TBIL) | 1966 | -0.0297 | [-0.0461, -0.0157] | 28.7 |
| Full cohort | ALT < 40 U/L | NLR → ALP → Lumbar spine | M1 (same-sample baseline) | 1966 | -0.0276 | [-0.0475, -0.0113] | 35.9 |
| Full cohort | ALT < 40 U/L | NLR → ALP → Lumbar spine | M3 (+ALT+GGT+TBIL) | 1966 | -0.0248 | [-0.0427, -0.0110] | 33.2 |
| Full cohort | ALP ≤ 125 U/L and ALT < 40 U/L | ALB → ALP → Femoral neck | M1 (same-sample baseline) | 1933 | 0.0053 | [-0.0008, 0.0112] | 12.0 |
| Full cohort | ALP ≤ 125 U/L and ALT < 40 U/L | ALB → ALP → Femoral neck | M3 (+ALT+GGT+TBIL) | 1933 | 0.0050 | [-0.0013, 0.0115] | 11.6 |
| Full cohort | ALP ≤ 125 U/L and ALT < 40 U/L | ALB → ALP → Lumbar spine | M1 (same-sample baseline) | 1933 | 0.0047 | [-0.0002, 0.0107] | 13.1 |
| Full cohort | ALP ≤ 125 U/L and ALT < 40 U/L | ALB → ALP → Lumbar spine | M3 (+ALT+GGT+TBIL) | 1933 | 0.0043 | [-0.0012, 0.0096] | 11.6 |
| Full cohort | ALP ≤ 125 U/L and ALT < 40 U/L | NLR → ALP → Femoral neck | M1 (same-sample baseline) | 1933 | -0.0183 | [-0.0309, -0.0102] | 18.3 |
| Full cohort | ALP ≤ 125 U/L and ALT < 40 U/L | NLR → ALP → Femoral neck | M3 (+ALT+GGT+TBIL) | 1933 | -0.0191 | [-0.0316, -0.0101] | 19.0 |
| Full cohort | ALP ≤ 125 U/L and ALT < 40 U/L | NLR → ALP → Lumbar spine | M1 (same-sample baseline) | 1933 | -0.0164 | [-0.0264, -0.0084] | 23.0 |
| Full cohort | ALP ≤ 125 U/L and ALT < 40 U/L | NLR → ALP → Lumbar spine | M3 (+ALT+GGT+TBIL) | 1933 | -0.0168 | [-0.0279, -0.0088] | 24.8 |
| Full cohort | ALP 45–125 U/L | ALB → ALP → Femoral neck | M1 (same-sample baseline) | 1777 | 0.0047 | [0.0013, 0.0096] | 7.7 |
| Full cohort | ALP 45–125 U/L | ALB → ALP → Femoral neck | M3 (+ALT+GGT+TBIL) | 1777 | 0.0040 | [-0.0001, 0.0086] | 6.3 |
| Full cohort | ALP 45–125 U/L | ALB → ALP → Lumbar spine | M1 (same-sample baseline) | 1777 | 0.0051 | [0.0010, 0.0094] | 11.8 |
| Full cohort | ALP 45–125 U/L | ALB → ALP → Lumbar spine | M3 (+ALT+GGT+TBIL) | 1777 | 0.0043 | [-0.0002, 0.0090] | 8.8 |
| Full cohort | ALP 45–125 U/L | NLR → ALP → Femoral neck | M1 (same-sample baseline) | 1777 | -0.0113 | [-0.0209, -0.0051] | 12.8 |
| Full cohort | ALP 45–125 U/L | NLR → ALP → Femoral neck | M3 (+ALT+GGT+TBIL) | 1777 | -0.0124 | [-0.0220, -0.0053] | 14.5 |
| Full cohort | ALP 45–125 U/L | NLR → ALP → Lumbar spine | M1 (same-sample baseline) | 1777 | -0.0126 | [-0.0218, -0.0052] | 18.4 |
| Full cohort | ALP 45–125 U/L | NLR → ALP → Lumbar spine | M3 (+ALT+GGT+TBIL) | 1777 | -0.0135 | [-0.0246, -0.0061] | 21.1 |
| Full cohort | ALT < 50 U/L | ALB → ALP → Femoral neck | M1 (same-sample baseline) | 2040 | 0.0068 | [0.0017, 0.0130] | 14.2 |
| Full cohort | ALT < 50 U/L | ALB → ALP → Femoral neck | M3 (+ALT+GGT+TBIL) | 2040 | 0.0052 | [-0.0004, 0.0103] | 11.2 |
| Full cohort | ALT < 50 U/L | ALB → ALP → Lumbar spine | M1 (same-sample baseline) | 2040 | 0.0062 | [0.0013, 0.0114] | 17.8 |
| Full cohort | ALT < 50 U/L | ALB → ALP → Lumbar spine | M3 (+ALT+GGT+TBIL) | 2040 | 0.0045 | [-0.0002, 0.0094] | 12.9 |
| Full cohort | ALT < 50 U/L | NLR → ALP → Femoral neck | M1 (same-sample baseline) | 2040 | -0.0261 | [-0.0422, -0.0136] | 25.1 |
| Full cohort | ALT < 50 U/L | NLR → ALP → Femoral neck | M3 (+ALT+GGT+TBIL) | 2040 | -0.0248 | [-0.0393, -0.0134] | 23.4 |
| Full cohort | ALT < 50 U/L | NLR → ALP → Lumbar spine | M1 (same-sample baseline) | 2040 | -0.0247 | [-0.0441, -0.0104] | 31.6 |
| Full cohort | ALT < 50 U/L | NLR → ALP → Lumbar spine | M3 (+ALT+GGT+TBIL) | 2040 | -0.0220 | [-0.0411, -0.0101] | 29.8 |
| Postmenopausal | ALP ≤ 125 U/L | ALB → ALP → Femoral neck | M1 (same-sample baseline) | 1222 | 0.0044 | [-0.0017, 0.0117] | 36.3 |
| Postmenopausal | ALP ≤ 125 U/L | ALB → ALP → Femoral neck | M3 (+ALT+GGT+TBIL) | 1222 | 0.0022 | [-0.0043, 0.0091] | 13.4 |
| Postmenopausal | ALP ≤ 125 U/L | ALB → ALP → Lumbar spine | M1 (same-sample baseline) | 1222 | 0.0041 | [-0.0015, 0.0100] | 85.5 |
| Postmenopausal | ALP ≤ 125 U/L | ALB → ALP → Lumbar spine | M3 (+ALT+GGT+TBIL) | 1222 | 0.0020 | [-0.0045, 0.0085] | 13.1 |
| Postmenopausal | ALP ≤ 125 U/L | NLR → ALP → Femoral neck | M1 (same-sample baseline) | 1222 | -0.0183 | [-0.0329, -0.0079] | 21.7 |
| Postmenopausal | ALP ≤ 125 U/L | NLR → ALP → Femoral neck | M3 (+ALT+GGT+TBIL) | 1222 | -0.0189 | [-0.0332, -0.0084] | 23.2 |
| Postmenopausal | ALP ≤ 125 U/L | NLR → ALP → Lumbar spine | M1 (same-sample baseline) | 1222 | -0.0169 | [-0.0329, -0.0055] | 19.8 |
| Postmenopausal | ALP ≤ 125 U/L | NLR → ALP → Lumbar spine | M3 (+ALT+GGT+TBIL) | 1222 | -0.0178 | [-0.0330, -0.0072] | 24.5 |
| Postmenopausal | ALT < 40 U/L | ALB → ALP → Femoral neck | M1 (same-sample baseline) | 1129 | 0.0050 | [-0.0031, 0.0149] | 45.4 |
| Postmenopausal | ALT < 40 U/L | ALB → ALP → Femoral neck | M3 (+ALT+GGT+TBIL) | 1129 | 0.0045 | [-0.0051, 0.0142] | 51.7 |
| Postmenopausal | ALT < 40 U/L | ALB → ALP → Lumbar spine | M1 (same-sample baseline) | 1129 | 0.0043 | [-0.0027, 0.0118] | 132.0 |
| Postmenopausal | ALT < 40 U/L | ALB → ALP → Lumbar spine | M3 (+ALT+GGT+TBIL) | 1129 | 0.0036 | [-0.0043, 0.0108] | 81.9 |
| Postmenopausal | ALT < 40 U/L | NLR → ALP → Femoral neck | M1 (same-sample baseline) | 1129 | -0.0278 | [-0.0464, -0.0133] | 31.3 |
| Postmenopausal | ALT < 40 U/L | NLR → ALP → Femoral neck | M3 (+ALT+GGT+TBIL) | 1129 | -0.0249 | [-0.0420, -0.0114] | 27.0 |
| Postmenopausal | ALT < 40 U/L | NLR → ALP → Lumbar spine | M1 (same-sample baseline) | 1129 | -0.0231 | [-0.0417, -0.0107] | 24.4 |
| Postmenopausal | ALT < 40 U/L | NLR → ALP → Lumbar spine | M3 (+ALT+GGT+TBIL) | 1129 | -0.0200 | [-0.0375, -0.0083] | 22.5 |
| Postmenopausal | ALP ≤ 125 U/L and ALT < 40 U/L | ALB → ALP → Femoral neck | M1 (same-sample baseline) | 1111 | 0.0042 | [-0.0036, 0.0125] | 36.6 |
| Postmenopausal | ALP ≤ 125 U/L and ALT < 40 U/L | ALB → ALP → Femoral neck | M3 (+ALT+GGT+TBIL) | 1111 | 0.0037 | [-0.0042, 0.0120] | 38.1 |
| Postmenopausal | ALP ≤ 125 U/L and ALT < 40 U/L | ALB → ALP → Lumbar spine | M1 (same-sample baseline) | 1111 | 0.0036 | [-0.0023, 0.0126] | 46.8 |
| Postmenopausal | ALP ≤ 125 U/L and ALT < 40 U/L | ALB → ALP → Lumbar spine | M3 (+ALT+GGT+TBIL) | 1111 | 0.0030 | [-0.0037, 0.0108] | 30.3 |
| Postmenopausal | ALP ≤ 125 U/L and ALT < 40 U/L | NLR → ALP → Femoral neck | M1 (same-sample baseline) | 1111 | -0.0224 | [-0.0406, -0.0094] | 26.5 |
| Postmenopausal | ALP ≤ 125 U/L and ALT < 40 U/L | NLR → ALP → Femoral neck | M3 (+ALT+GGT+TBIL) | 1111 | -0.0210 | [-0.0393, -0.0076] | 24.1 |
| Postmenopausal | ALP ≤ 125 U/L and ALT < 40 U/L | NLR → ALP → Lumbar spine | M1 (same-sample baseline) | 1111 | -0.0193 | [-0.0360, -0.0079] | 21.6 |
| Postmenopausal | ALP ≤ 125 U/L and ALT < 40 U/L | NLR → ALP → Lumbar spine | M3 (+ALT+GGT+TBIL) | 1111 | -0.0172 | [-0.0311, -0.0058] | 21.2 |
| Postmenopausal | ALP 45–125 U/L | ALB → ALP → Femoral neck | M1 (same-sample baseline) | 1056 | 0.0039 | [-0.0015, 0.0107] | 19.2 |
| Postmenopausal | ALP 45–125 U/L | ALB → ALP → Femoral neck | M3 (+ALT+GGT+TBIL) | 1056 | 0.0018 | [-0.0034, 0.0085] | 7.1 |
| Postmenopausal | ALP 45–125 U/L | ALB → ALP → Lumbar spine | M1 (same-sample baseline) | 1056 | 0.0046 | [-0.0017, 0.0118] | 51.3 |
| Postmenopausal | ALP 45–125 U/L | ALB → ALP → Lumbar spine | M3 (+ALT+GGT+TBIL) | 1056 | 0.0021 | [-0.0052, 0.0086] | 10.4 |
| Postmenopausal | ALP 45–125 U/L | NLR → ALP → Femoral neck | M1 (same-sample baseline) | 1056 | -0.0151 | [-0.0317, -0.0050] | 21.7 |
| Postmenopausal | ALP 45–125 U/L | NLR → ALP → Femoral neck | M3 (+ALT+GGT+TBIL) | 1056 | -0.0154 | [-0.0317, -0.0054] | 23.6 |
| Postmenopausal | ALP 45–125 U/L | NLR → ALP → Lumbar spine | M1 (same-sample baseline) | 1056 | -0.0177 | [-0.0338, -0.0065] | 19.8 |
| Postmenopausal | ALP 45–125 U/L | NLR → ALP → Lumbar spine | M3 (+ALT+GGT+TBIL) | 1056 | -0.0184 | [-0.0344, -0.0068] | 23.9 |
| Postmenopausal | ALT < 50 U/L | ALB → ALP → Femoral neck | M1 (same-sample baseline) | 1168 | 0.0036 | [-0.0040, 0.0117] | 47.9 |
| Postmenopausal | ALT < 50 U/L | ALB → ALP → Femoral neck | M3 (+ALT+GGT+TBIL) | 1168 | 0.0025 | [-0.0066, 0.0123] | 35.9 |
| Postmenopausal | ALT < 50 U/L | ALB → ALP → Lumbar spine | M1 (same-sample baseline) | 1168 | 0.0032 | [-0.0048, 0.0103] | 65.1 |
| Postmenopausal | ALT < 50 U/L | ALB → ALP → Lumbar spine | M3 (+ALT+GGT+TBIL) | 1168 | 0.0022 | [-0.0064, 0.0100] | 26.8 |
| Postmenopausal | ALT < 50 U/L | NLR → ALP → Femoral neck | M1 (same-sample baseline) | 1168 | -0.0258 | [-0.0449, -0.0122] | 29.0 |
| Postmenopausal | ALT < 50 U/L | NLR → ALP → Femoral neck | M3 (+ALT+GGT+TBIL) | 1168 | -0.0233 | [-0.0392, -0.0103] | 25.5 |
| Postmenopausal | ALT < 50 U/L | NLR → ALP → Lumbar spine | M1 (same-sample baseline) | 1168 | -0.0228 | [-0.0431, -0.0110] | 24.7 |
| Postmenopausal | ALT < 50 U/L | NLR → ALP → Lumbar spine | M3 (+ALT+GGT+TBIL) | 1168 | -0.0200 | [-0.0346, -0.0087] | 23.4 |

*Subgroups were defined using raw (non-imputed) laboratory values: ALP ≤ 125 U/L (upper limit of the normal range); ALT < 40 U/L; both ALP ≤ 125 U/L and ALT < 40 U/L; ALP 45–125 U/L (strict normal range); and ALT < 50 U/L (robustness check). For each subgroup, the base model (M1: covariates age and menopause status in the full cohort, age in the postmenopausal subgroup) and the fully adjusted model (M3: additionally adjusting for ALT, GGT, and total bilirubin) were run on the identical same-sample baseline sample. IE: indirect effect; CI: 95% bootstrap confidence interval; PM: proportion mediated; DE/TE: direct/total effect. Statistical significance was defined as a bootstrap 95% CI excluding zero.*
